# Post-operative Horner’s Syndrome: A Rare Complication Following Posterior Scoliosis Surgery in a Patient With Syringomyelia

**DOI:** 10.7759/cureus.25242

**Published:** 2022-05-23

**Authors:** Isaac J May, Andrew J Berg, David Dillon

**Affiliations:** 1 General Medicine, Royal Perth Hospital, Perth, AUS; 2 State Spinal & Scoliosis Service, Royal Perth Hospital, Perth, AUS

**Keywords:** spinal surgery, paediatric surgery, posterior spinal fixation and fusion, scoliosis surgery, syringomyelia, horner’s syndrome

## Abstract

Horner’s syndrome following posterior spinal instrumentation for scoliosis has been rarely reported. We describe the case of a 15-year-old male who presented with right-sided ptosis, miosis, and anhidrosis after scoliosis correction. This is the first reported case of first-order Horner’s syndrome developing after scoliosis repair via posterior fixation in a patient known to have asymptomatic syringomyelia. The impression was that Horner’s syndrome developed secondary to increased traction of the syringomyelia after scoliosis repair. This is significant as a diagnosis of Horner’s syndrome can be distressing to patients and chronic cases cause cosmetic defects that might require surgical correction. We suggest that similar patients should be warned pre-operatively given the psychological distress associated with chronic Horner's syndrome. This case also illustrates the importance of an appropriate workup to rule out other sinister pathologies that can cause Horner’s syndrome.

## Introduction

Horner’s syndrome results from disruption of the sympathetic nervous system, presenting with the classic triad of ptosis, anhidrosis, and miosis. There are numerous potential causes for Horner’s syndrome, which can be classified as first-, second-, or third-order lesions depending on the location of the pathology. A first-order Horner's syndrome occurs when there is a disruption of the first-order neurons descending from the hypothalamus down to the level of C8-T2 (ciliospinal centre of Budge and Waller). Spinal cord lesions above the ciliospinal centre such as vascular malformations, malignancies, infarction, and syringomyelia are also first-order pathologies. Second-order Horner's syndrome occurs when there is disruption of the preganglionic neurons exiting from the ciliospinal centre, which run along the pulmonary apex to the superior cervical ganglion. Third-order Horner's syndrome occurs when there is a disruption of the postganglionic neurons, which run along the carotid artery and into the cavernous sinus, before joining with the ophthalmic branch of the trigeminal nerve. Unlike first- and second-order Horner's syndrome, third-order Horner's syndrome does not present with anhidrosis [1].

Surgical procedures of the neck, chest, skull base, and paraspinal region have been identified as common causes [2]. Some serious pathologies that can present with Horner's syndrome include an apical lung tumour and carotid artery dissection (second- and third-order causes) [2-4]. Horner's syndrome is an uncommon complication of anterior cervical spinal surgery (0.06% of 8887 patients in a multicentre study) [5], and it is an even rarer complication of posterior spinal procedures with only a handful of cases reported [6-8]. Furthermore, it may develop post-surgically in patients with idiopathic syringomyelia without an associated Chiari malformation, a rare complication that has only been documented in a few cases reports but not following posterior spinal surgery [9-13]. Here we report on a case, which is the first known incident of a first-order Horner's syndrome developing after posterior spinal surgery in a patient with a previously known asymptomatic idiopathic syringomyelia. We suspect these patients may be at increased risk of developing Horner's syndrome.

## Case presentation

**Figure 1:**
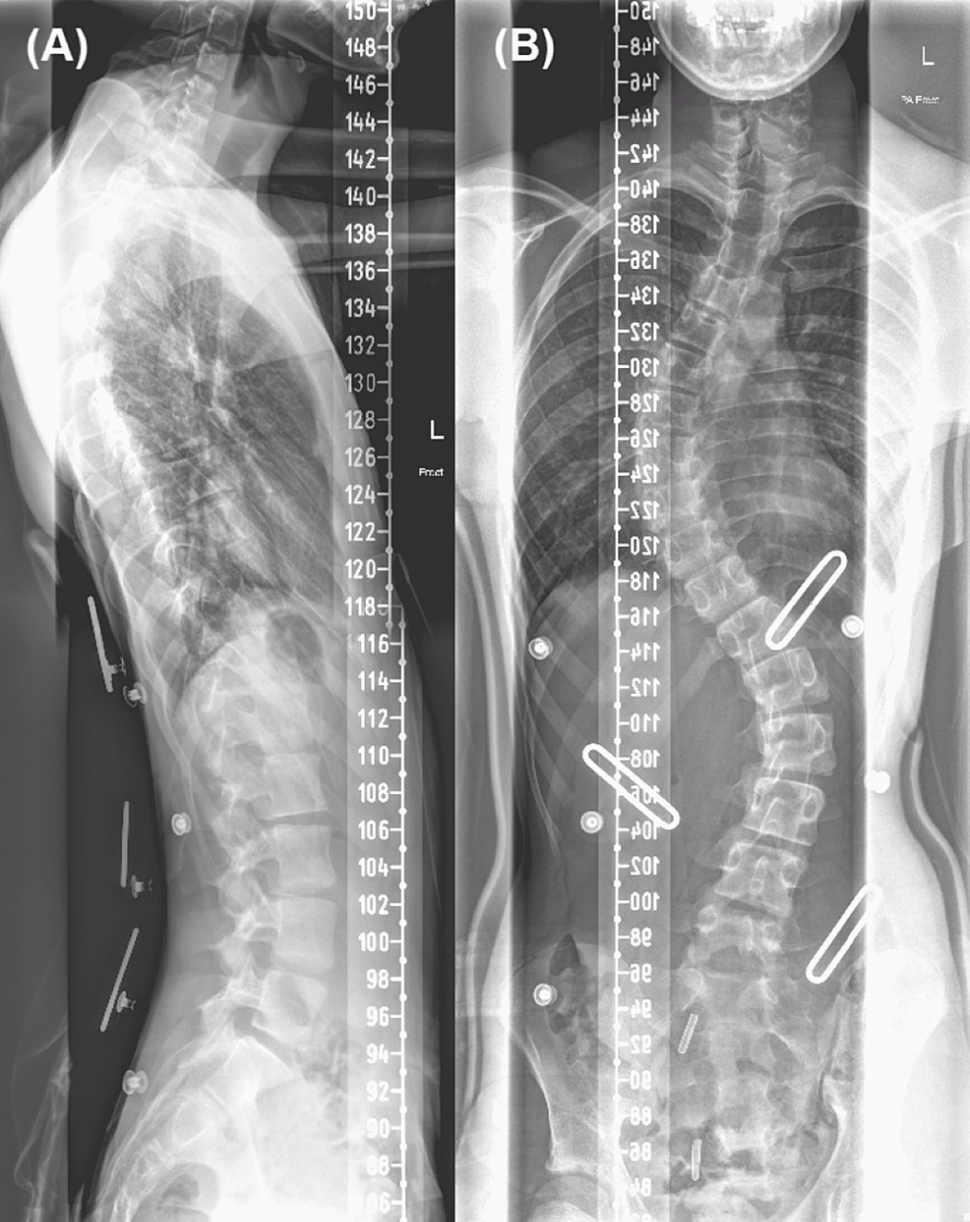
Pre-operative scoliogram. (A) Lateral view. (B) AP view. AP, anteroposterior.

**Figure 2:**
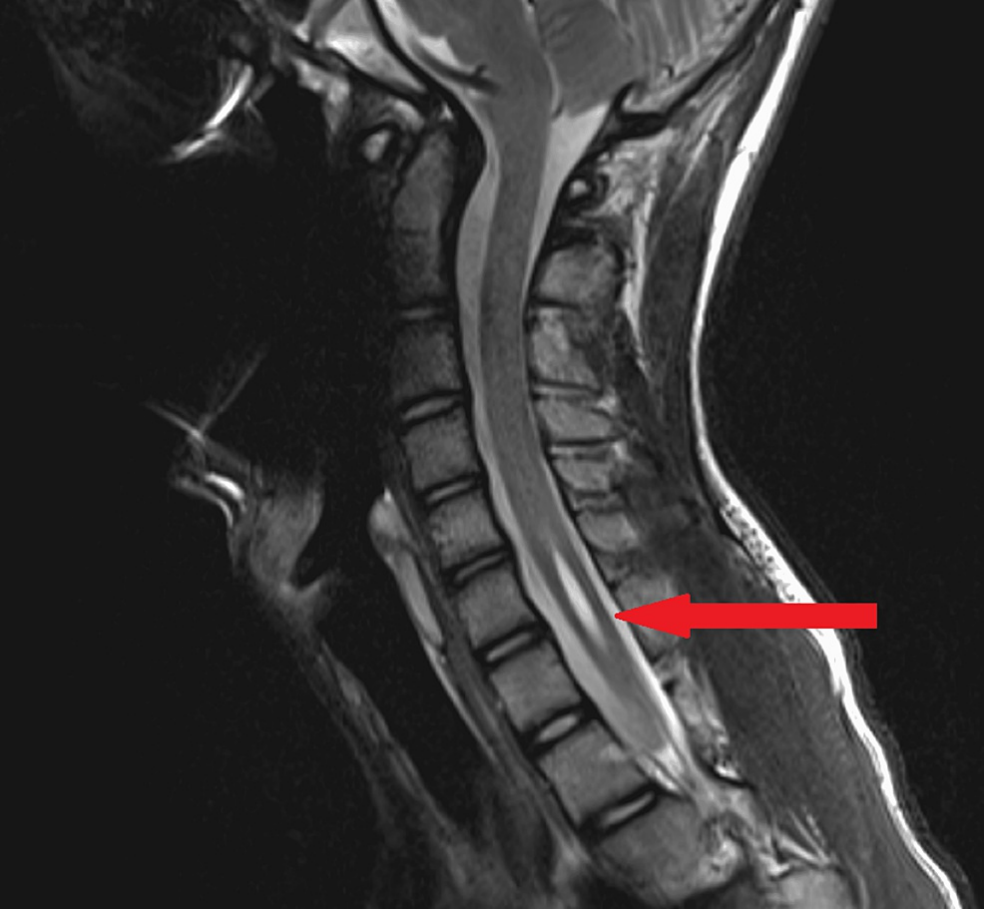
Pre-operative MRI spine. Note the short segment syringomyelia. It extends from the level of the C5/6 disc space to the inferior end-plate of C7, superior to the level of the ciliospinal centre of Budge and Waller.

**Figure 3:**
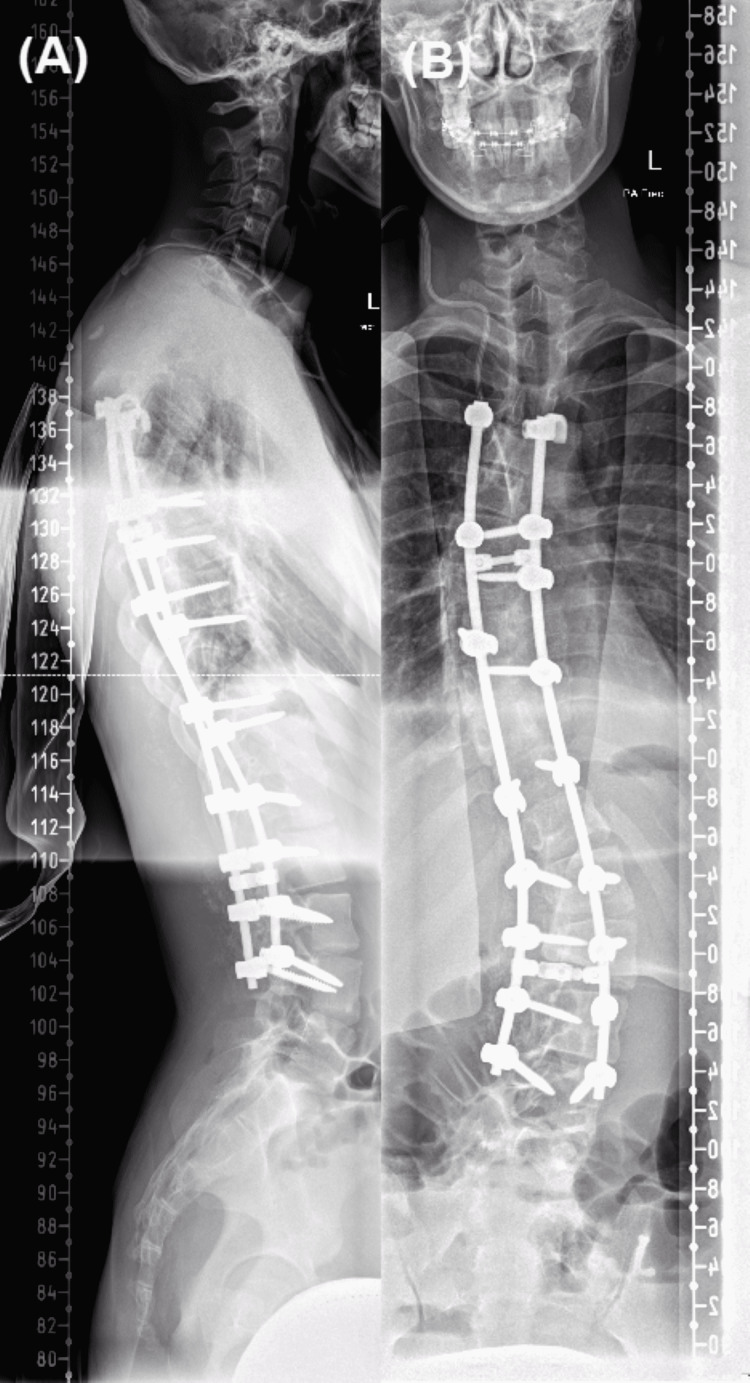
Post-operative scoliogram showing adequate vertebral alignment. (A) Lateral view. (B) AP view. AP, anteroposterior.

A 15-year-old male presented for elective surgery for correction of his scoliosis, undergoing a posterior fixation of T4 to L4. Pre-operatively he had a rightward convexity centred at the T8/9 level with Cobb’s angle of 57 degrees, and a leftward convexity centred at the L2/3 level with Cobb’s angle of 48 degrees (Figure 1). An MRI had identified asymptomatic syringomyelia without a Chiari malformation in the cervical spine (Figure 2). He had no other known medical conditions. A post-operative scoliogram showed adequate correction of scoliosis (Figure 3).

**Figure 4:**
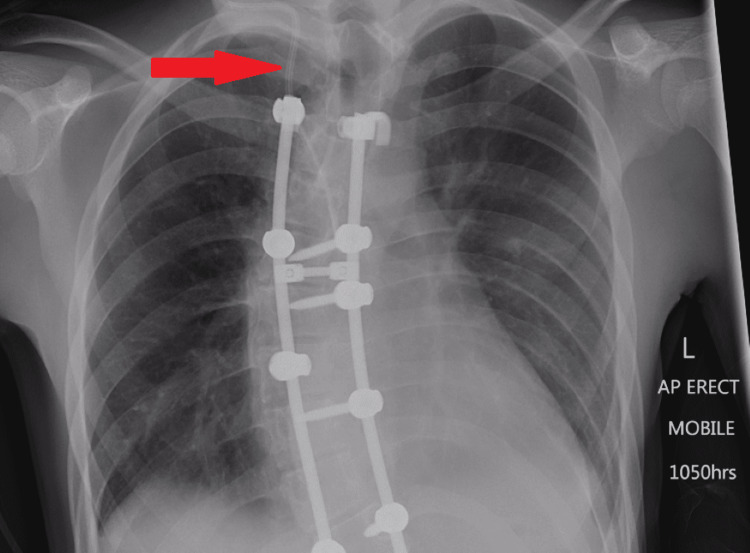
Chest X-ray performed for septic screen. The internal jugular vein central venous catheter is visible as indicated by the arrow.

An internal jugular vein (IJV) central venous catheter was inserted after surgery (Figure 4). Post-operatively he experienced intermittent paraesthesia in his left thumb (C6 dermatome) which resolved on day 2. The patient was not given epidural anaesthesia for pain relief, and instead received a ketamine infusion which ceased on day 3. He had a single temperature spike on day 3, for which a septic screen (blood cultures, chest X-ray, urine microscopy and culture) yielded no abnormalities. He remained haemodynamically stable for the rest of his admission. The patient was not commenced on post-operative deep vein thrombosis prophylaxis, given the high risk of haematoma development and because he was expected to begin mobilizing fairly soon in his recovery. The patient received physiotherapy input, mobilized well, and was discharged on day 6 of his admission.

He represented four days after discharge for progressively worsening right-sided ptosis, miosis, and anhidrosis. He had not experienced any blunt trauma to his neck and denied any headaches. He did not report any other symptoms. Other than the triad of signs described, the remainder of his neurological examination was normal. His other system examinations and vitals were also normal. No paediatric or adult malignancies were reported in his family history, nor was there any reported history of strokes.

The patient was diagnosed with Horner's syndrome. Chest X-rays during his recent admission performed as part of his septic screen and from his scoliogram (Figures 3, 4) showed no evidence of lesions within the apices of the lungs. Given his recent scoliosis surgery, his young age, and the acute onset of Horner's syndrome, the initial concern was that he may have developed a carotid artery dissection or epidural haematoma. Investigations were targeted to rule out these pathologies.

An MRI of the brain and spine was performed to investigate carotid artery dissection and epidural haematoma, but the study was technically limited due to the early termination of the scan by the patient. The MRI visualized his previously known syringomyelia and did not identify any evidence of an epidural haematoma. The syringomyelia was reported to be stable in size relative to his previous MRI at the level of the image's resolution. Assessment of the upper cervical region was limited by artefact, and thus could not rule out a carotid artery dissection.

**Figure 5:**
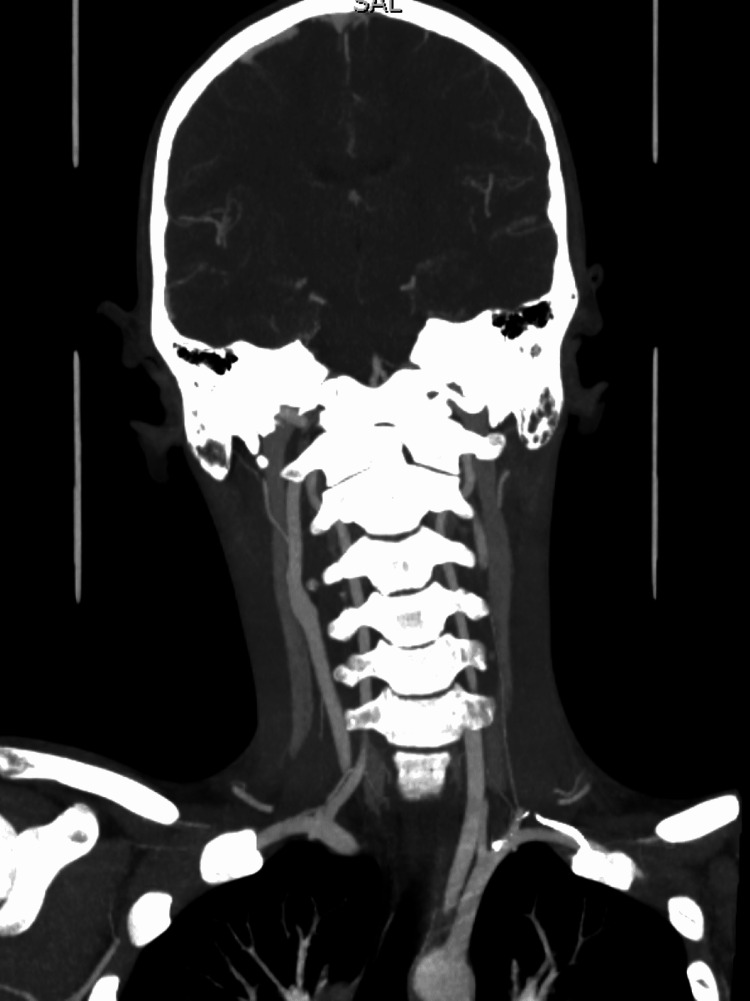
CT angiogram of head and neck. No carotid artery dissection was identified.

A CT angiogram of the head and neck was performed due to the inadequacy of the MRI. It showed normal opacification of the carotid arteries without evidence of dissection, and no evidence of intracranial aneurysms or other vascular malformations in the neck (Figure 5). His lung apices were further confirmed to be clear on the CT scan with no evidence of any apical lesions that could have been disrupting the sympathetic chain.

These investigations ruled out carotid artery dissection and epidural haematoma, the two main sinister pathologies after spinal surgery that may have caused Horner's syndrome to develop. As neither sinister pathology was identified, the patient was discharged with ongoing outpatient follow-up under neurology and spinal surgery specialties. The patient did not develop any further complications, and one month after onset the patient only had mild anisocoria (right pupil 1 mm smaller than the left), but no other neurological signs or symptoms. Six months after surgery the patient was reviewed as part of regular spinal patient follow-up, confirming complete resolution of his Horner's syndrome with no additional complications.

## Discussion

Horner’s syndrome as a complication of scoliosis repair is a novel finding because there is little reported in the literature of it occurring after scoliosis repair by posterior correction. Cowie et al. reported a case in which an 11-year-old girl developed a left-sided Horner's syndrome preceded by left-sided C8 paraesthesia secondary to epidural anaesthesia after scoliosis repair (T4-L1 fusion). Horner's syndrome was detected 24 hours after surgery. The epidural rate was weaned over 16 hours, and Horner's syndrome resolved within this time frame [6]. Similarities with this case are that both patients presented with cervical dermatomal paraesthesia before the onset of Horner’s syndrome. However, epidural anaesthesia was not used in our case, and in Cowie et al.’s there was no history of syringomyelia. Hered et al. also reported a case in which Horner’s syndrome was detected three days after scoliosis repair with epidural analgesia. In this case, it did not resolve and required surgical correction for the patient’s ptosis, demonstrating that this complication can have serious long-term effects [7].

Mueller et al. are purported to have been the first to document this sequela - a case of Horner’s syndrome developing after posterior fusion without the use of epidural anaesthesia reported two weeks after surgery. They postulated that it was a second-order Horner's syndrome (preganglionic) that may have been caused by trauma to the T4 pedicle intraoperatively [8].

Bali reported a case in which Horner's syndrome was detected 24 hours post-operatively. They suggested but could not confirm that it may have developed secondary to either a complicated IJV catheterization requiring multiple attempts or from the patient being in the prone position. They reported a normal MRI, which did not identify a jugular vein haematoma or injury to the internal carotid artery [14]. IJV catheterization has been implicated as a cause of Horner's syndrome in a number of case reports, and identified direct trauma to the carotid as well as sympathetic chain compression from jugular vein haematomas as causes in some cases [15]. Similarly, our patient also underwent IJV catheterization. While we acknowledge it as a possible cause, we think it is less likely as the Horner's syndrome would theoretically be a third-order one due to the lesions' location, and therefore would not present with anhidrosis [1]. Furthermore, the MRI and CT angiogram showed no evidence of haematoma or other vascular injuries secondary to IJV catheterization.

None of these cases reported a history of syringomyelia. Horner's syndrome in association with syringomyelia has been reported as causing Horner's syndrome in a few cases but without a history of posterior spinal surgery [9-13].

In all of the cases discussed, the detection of Horner's syndrome was delayed. The earliest was reported at 24 hours, and the latest at two weeks [6-8]. Hered et al. reported the patient had facial oedema post-operatively and suspected that it had masked their Horner's syndrome and prevented early identification [7]. Mueller et al. reported a collateral history from the patient's mother after discharge in which she stated that she noticed ptosis after the patient's eyelids became less swollen. They did not report detecting facial oedema during the admission, but noted on review of the fluid balance record that the patient was overloaded by 851 ml on discharge. Unfortunately, the presence or absence of facial oedema post-operatively in this case was not recorded, and we, therefore, cannot assume that Horner's syndrome was masked by this resulting in delayed diagnosis. However, given that facial oedema was identified in these other cases and is a known complication of surgery in the prone position [16], it is possible that the delayed presentation of Horner's syndrome, in this case, could have been due to a delayed diagnosis secondary to facial oedema.

In this case, after reviewing by neurologists, it was suggested that Horner's syndrome developed secondary to the surgery causing transient alteration of the dynamics of his lower cervical syringomyelia. Although the repeat MRI of the patient’s spinal cord showed the syringomyelia had not changed in size, the syringomyelia may also have stretched to a degree that was imperceptible to the radiologist on the imaging’s resolution. This could have been complicated by artefact from the poor technical quality of the scan due to its early termination. A syringomyelia would already cause a small degree of traction/stretching on the surrounding nerves, so further compounding traction from the surgery could have resulted in disruption of the hypothalamospinal tract. This would, therefore, classify as a first-order Horner's syndrome due to the location of the syringomyelia at C5-C7 (within the spinal cord above the level of the ciliospinal centre of Budge and Waller).

## Conclusions

In conclusion, this is the first reported case of Horner’s syndrome developing after scoliosis repair in a patient with known syringomyelia. We suggest that patients with a syringomyelia undergoing scoliosis correction via posterior fixation may be at increased risk of developing Horner's syndrome. We also acknowledge from our review of the literature that epidural anaesthesia and IJV catheterization are both risk factors for developing Horner's syndrome. While IJV catheterization is a possible differential for the cause of this case's Horner's syndrome, we suspect it is less likely due to the presence of anhidrosis and because MRI and CT angiogram of the neck were normal. 

Also of significance is that facial oedema has in previous cases masked post-operative Horner's syndrome causing delayed diagnosis. While we cannot confirm if facial oedema was the cause of delayed diagnosis in this case, we recommend that patients after spinal surgery be examined specifically for Horner's syndrome and facial oedema should be accounted for. This could allow for Horner's syndrome to be diagnosed sooner post-operatively, which may assist in identifying the causative pathology.

Finally, we recommend that patients with syringomyelia who require scoliosis correction be warned pre-operatively of Horner's syndrome as a potential complication. This is due to the potential long-term effects Horner's syndrome can have on the patient, as seen in cases with poor prognosis where surgical correction was required.
